# DNA methylation markers detected in blood, stool, urine, and tissue in colorectal cancer: a systematic review of paired samples

**DOI:** 10.1007/s00384-020-03757-x

**Published:** 2020-10-06

**Authors:** Eivor Alette Laugsand, Siv Sellæg Brenne, Frank Skorpen

**Affiliations:** 1 Department of Surgery, Levanger Hospital, Nord-Trøndelag Hospital trust, N-7600 Levanger, Norway; 2 Department of Public Health and Nursing, Faculty of Medicine and Health Sciences, Norwegian University of Science and Technology (NTNU), N-7491 Trondheim, Norway; 3 Department of Clinical and Molecular Medicine, Faculty of Medicine and Health Sciences, Norwegian University of Science and Technology (NTNU), N-7491 Trondheim, Norway

**Keywords:** Colorectal cancer, Liquid biopsy, DNA methylation, cfDNA, Biomarker

## Abstract

**Purpose:**

Methylated cell-free DNA in liquid biopsies are promising non-invasive biomarkers for colorectal cancer (CRC). Optimal markers would have high sensitivity and specificity for early detection of CRC and could be detected in more than one type of material from the patient. We systematically reviewed the literature on DNA methylation markers of colorectal cancer, detected in more than one type of material, regarding their potential as contributors to a panel for screening and follow-up of CRC.

**Methods:**

The databases MEDLINE, Web of Science, and Embase were systematically searched. Data extraction and review was performed by two authors independently. Agreement between methylation status in tissue and other materials (blood/stool/urine) was analyzed using the McNemar test and Cohen’s kappa.

**Results:**

From the 51 included studies, we identified seven single markers with sensitivity ≥ 75% and specificity ≥ 90% for CRC. We also identified one promising plasma panel and two stool panels. The correspondence of methylation status was evaluated as very good for four markers, but only marginal for most of the other markers investigated (12 of 21).

**Conclusion:**

The included studies reported only some of the variables and markers of interest and included few patients. Hence, a meta-analysis was not possible at this point. Larger, prospective studies must be designed to study the discordant detection of markers in tissue and liquid biopsies. When reporting their findings, such studies should use a standardized format.

**Electronic supplementary material:**

The online version of this article (10.1007/s00384-020-03757-x) contains supplementary material, which is available to authorized users.

## Introduction

Colorectal cancer (CRC) develops over years or decades through genetic and epigenetic alterations [1]. CRC is the second leading cause of cancer-related death in the Western world [2, 3], and its treatment and follow-up have a massive impact on the quality of life [4, 5]. Measures to increase CRC survival would be to diagnose the disease at an earlier stage, more reliably identify patients with residual disease after treatment, more accurately diagnose recurrence, and more closely monitor the effect of oncologic treatment. Sensitive and reliable biomarkers in blood, stool, or urine would be ideal for this purpose. Hence, much effort has been put into the identification of new and improved biomarkers for early detection and follow-up of CRC [6, 7]. Cell-free DNA (cfDNA) methylation markers, especially in blood and stool, are considered promising biomarkers, with remarkably high sensitivity and specificity for CRC [8, 9].

Methylation of cytosine to form 5-methylcytosine at CpG dinucleotides is a widespread and normal epigenetic modification of the DNA in humans. Increased CpG methylation in promoter regions of genes, especially at CpG-rich sequences termed CpG islands, is associated with transcription repression [10]. Increased methylation in classical tumor suppressor genes, genes regulating mitosis, and DNA repair is considered an early event in CRC tumorigenesis [1, 11]. Many individual markers have been investigated to date [11–16]. However, it is considered that the most useful tool for detection and follow-up of CRC would be a panel of cell-free DNA markers with high sensitivity and specificity for early detection of the disease, with prognostic value, with the possibility to detect residual disease, and recurrence, as well as ability to change as a result of oncologic treatment [17]. Detection even at the adenoma level would be preferable [18]. Also, optimal markers would be possible to detect both in the blood (^b^), stool (^s^), urine (^u^), and tumor tissue (^t^) throughout the course of the disease [19].

Much of the research on specific methylation markers of CRC has focused on tumor-derived DNA. Lately, single markers and panels of markers have also been tested in tumor remote media such as blood, stool, and urine and have gained much attention as potential liquid biopsies (non-invasive cancer biomarkers) [20, 21]. Somewhat challenging, detection of such markers in blood/urine/stool has not been concordant with detection in CRC tissue [22–24]. This inconsistency between liquid biopsies and tissue biopsies has been pointed out as one of the hurdles that need to be addressed before liquid biopsies can be taken into routine clinical use [25]. To overcome this issue, some studies have analyzed methylation markers in several different materials from the same individual. The present review focuses on analyses performed in more than one type of material from the same individual. The objective is to identify cell-free DNA methylation markers of colorectal cancer detected in more than one type of material from the same patient and systematically review their potential as contributors to a panel for screening and follow-up of CRC.

## Methods

The search strategy, study inclusion and exclusion criteria, data extraction, study quality assessment, and data analysis were performed based on the Preferred Reporting Items for Systematic Reviews and Meta-analyses (PRISMA), the variant for diagnostic test accuracy (PRISMA-DTA), and the quality assessment for diagnostic accuracy studies (QUADAS) [26–29].

### Search strategy, study inclusion, and exclusion

**Fig. 1:**
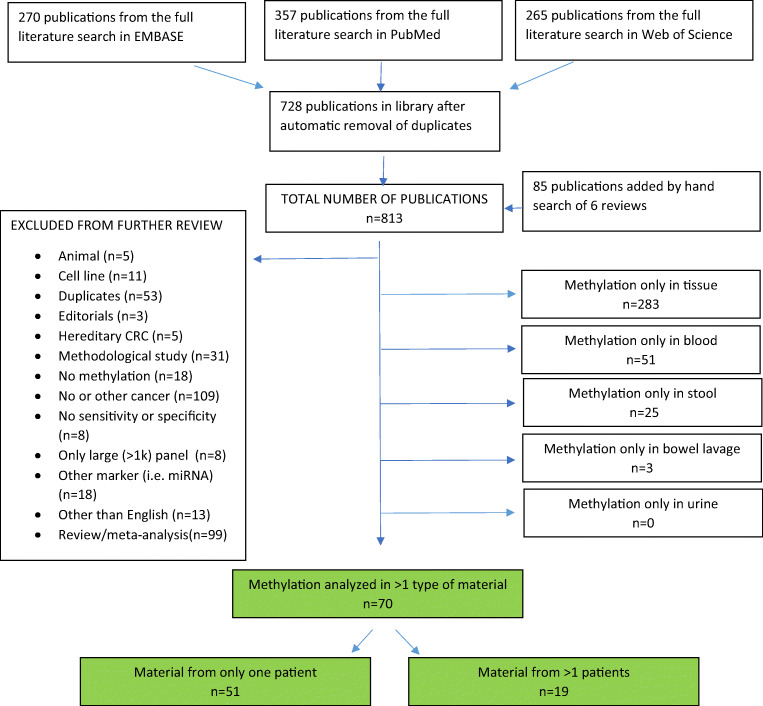
Flowchart for selection of publications

A systematic literature search was performed in MEDLINE, Web of Science, and Embase databases using the following search strategy: (1) «hypermethylation» or «methylation» or «hypermetylation» or «metylation» or «CpG islands», (2) «biomarker» or «liquid biopsy» or «non-invasive» or «ctDNA» or «cfDNA», (3) «colorectal cancer» or «colorectal neoplasm» or «adenoma», (4) («tumour» or «tumor» or «biopsy») or («blood» or «serum» or «plasma» or «blood analysis») or stool or urine, (5) «PCR» or «microarray» or «sequencing», (6) #1 AND #2 AND #3 AND #4 AND #5, (7) «animal» or «cell line», (8) #6 NOT #7. Studies were included if they described one or more DNA methylation markers in more than one type of material from the same individual (blood, stool, urine, or tumor samples) and correlated their finding with CRC. Six recent reviews were hand searched for publications not identified by the systematic search [11–16]. Reviews, case reports, and duplicates including conference abstracts later published as full text, studies on animal models or cell lines, as well as papers written in languages other than English were excluded. Study inclusion and exclusion criteria are shown in the PRISMA flowchart (Fig. 1).

### Data extraction

Data were extracted in a pre-defined form including the type of specimen, sensitivity for CRC (*n*/*N*, %), sensitivity for adenoma (*n*/*N*, %), specificity (*n*/*N*, %), and method for methylation analysis. Data extraction and review was performed by two authors independently.

### Statistical analysis

Agreement between methylation status in tissue and other materials (blood/stool/urine) was analyzed using the McNemar test and Cohen’s kappa. If the *p* value of the McNemar test was insignificant (*p* ≥ 0.05) and the *p* value of the Kappa statistic was significant (*p* < 0.05), the methylation status was considered as being in agreement. The criteria for the strength of agreement were as follows: *K* < 0.2 poor, *K* 0.21–0.40 fair, *K* 0.41–0.60 moderate, *K* 0.61–0.80 good, *K* 0.81–1.00 very good [30]. All statistics were performed using SPSS 25 for windows (SPSS Inc., Chicago, IL, USA)

## Results

The systematic literature search identified 728 publications of potential relevance and 85 publications were added by hand search. The abstracts of these 813 publications were reviewed and 381 publications were excluded. Among the remaining, 283 publications considered methylation only in tissue, 51 publications considered methylation only in the blood, 25 publications considered methylation only in stool, and three publications considered methylation only in the bowel lavage (Fig. 1). No publications considered urine only. Methylation in more than one type of material was analyzed in 70 publications, among which only 51 publications analyzed methylation in more than one type of material from the same individual.

The 51 included studies demonstrate that some of the well-known cell-free DNA methylation markers of colorectal cancer can be detected in more than one type of material from the same patient. The extracted data are presented in full as supplementary material (Supplementary Table 1 and 2). Single markers with sensitivity for CRC of 75% or above in tissue and at least one other material were *BMP3*^b^, *EFHD1*^b^, *ITGA4*^s^, *NDRG4*^s^, *OSMR*^b^ *PPP1R3C*^b^, *SEPT9*^b^, *SFRP1*^b^, *SFRP2*^s^, *SPG20*^b,s^, *TFPI2*^s^, and *VIM*^u^ (Table 1). Among these, *ITGA4*, *SEPT9*, *SFRP1*, *SFRP2*, *SPG20*, *TFPI2*, and *VIM* had a specificity of 90% or above (Table 1).

**Table 1:** Single markers with sensitivity for CRC of 75% or above, in tissue and at least one other material

*Gene*	Specimen	Sensitivity CRC % (*n*)	Sensitivity adenoma % (*n*)	Specificity % (*n*)	Method	Reference
*BMP3*	Plasma	75 (44/59)	No adenomas	70 (26/37)	MSP	[31]
*BMP3*	Tissue	81 (24 /30)	No adenomas	ns (ns/37)	MSP	[31]
*EFHD1*	Tissue	79 (19/24)	No adenomas	ns (ns/ns)	MSP	[32]
*EFHD1*	Plasma	79 (19/24)	No adenomas	78 (75/96)	MSP	[32]
*ITGA4*	Tissue	89 (8/9)	88 (44/50)	No controls	nMSP	[33]
*ITGA4*	Stool	80 (4/5)	No adenomas	100 (5/5)	nMSP	[33]
*NDRG4*	Tissue	81 (68/84)	No adenomas	92 (77/84)	nMSP	[34]
*NDRG4*	Stool	76 (64/84)	No adenomas	89 (ns/ns)	nMSP	[34]
*OSMR*	Plasma	75 (30/40)	No adenomas	No controls	MSP	[35]
*OSMR*	Tissue	95 (38/40)	No adenomas	No controls	MSP	[35]
*PPP1R3C*	Tissue	92 (22/24)	No adenomas	ns (ns/ns)	MSP	[32]
*PPP1R3C*	Plasma	79 (19/24)	No adenomas	81 (78/96)	MSP	[32]
*SEPT9*	Plasma	75 (136/182)	No adenomas	97 (164/170)	qMSP	[36]
*SEPT9*	Tissue	78 (99/127)	No adenomas	97 (116/120)	qMSP	[36]
*SEPT9*	Tissue	97 (33/34)	100 (26/26)	96 (23/24)	MSP	[37]
*SEPT9*	Plasma	88 (30/34)	31 (8/26)	92 (22/24)	MSP	[37]
*SFRP1*	Tissue	92 (23/25)	ns (ns/22)	ns (ns/56)	MSP	[38]
*SFRP1*	Plasma	80 (20/25)	17 (3/18)	92 (33/36)	MSP	[38]
*SFRP2*	Tissue	88 (149/169)	65 (41/63)	100 (30/30)	MSP	[39]
*SFRP2*	Stool	84 (142/169)	46 (29/63)	93 (28/30)	MSP	[39]
*SFRP2*	Serum	67 (113/169)	6 (4 /63)	100 (30/30)	MSP	[39]
*SFRP2*	Tissue	91 (63/69)	79 (27/34)	100 (30/30)	MSP	[40]
*SFRP2*	Stool	87 (60/69)	62 (21/34)	93 (28/30)	MSP	[40]
*SPG20*	Tissue	94 (30/32)	No adenomas	99 (ns/32)	qMSP	[41]
*SPG20*	Plasma	81 (30/37)	No adenomas	97 (ns/37)	qMSP	[41]
*SPG20*	Tissue	85 (82/96)	No adenomas	No controls	MSP	[42]
*SPG20*	Stool	80 (77/96)	No adenomas	100 (30/30)	MSP	[42]
*TFPI2*	Tissue	89 (8/9)	64 (32/50)	No controls	nMSP	[33]
*TFPI2*	Stool	80 (4/5)	No adenomas	100 (5/5)	nMSP	[33]
*TFPI2*	Tissue	99 (114/115)	98 (55/56)	94 (45/48)	MSP	[43]
*TFPI2*	Stool	76 (50/66)	21 (4/19)	93 (28/30)	qMSP	[43]
*VIM*	Tissue	85 (17/20)	No adenomas	ns (ns/20)	qMSP	[44]
*VIM*	Urine	75 (15/20)	No adenomas	90 (18/20)	qMSP	[44]

*ns*, not specified; *MSP*, methylation-specific PCR; *qMSP*, quantitative methylation-specific PCR; *nMSP*, nested methylation-specific PCR; *pSEQ*, pyrosequencing

The most promising markers tested as panels were the plasma panel *APC/MGMT/RASSF2A/Wif-1* (sensitivity 87%, specificity 92%) [45], the stool panels *BMP3/NDRG4/VIM/TFPI2*/mutant *KRAS*/*ACTB* (β-actin; used as reference gene for normalization purposes)/Hb (sensitivity 87%, specificity 93%) [46], and *RARB2/p16/MGMT/APC* (sensitivity 75%, specificity 100%) [47] (Table 2).

**Table 2:** Panel markers with sensitivity for CRC of 75% or above, in at least one type of material

*Genes*	Specimen	Sensitivity CRC % (*n*)	Sensitivity adenoma % (*n*)	Specificity % (*n*)	Method	Reference
*BMP3*, *NDRG4*, *VIM*, *TFPI2*, *mutant KRAS*, *B-actin*, Hb	Stool	87 (26/30)	82 (18/22)	93 (43/46)	QuARTS	[48]
*SEPT9*	Plasma	60 (18/30)	14 (3/22)	73 (36/49)	MSP	[48]
*p14*	Tissue	18 (ns/243)	not tested	98 (ns/148)	MSP	[45]
*p16*	Tissue	34 (ns/243)	not tested	97 (ns/148)	MSP	[45]
*APC*	Tissue	27 (ns/243)	18 (ns/64)	97 (ns/148)	MSP	[45]
*DAPK*	Tissue	34 (ns/243)	not tested	100 (148/148)	MSP	[45]
*HLTF*	Tissue	32 (ns/243)	not tested	98 (ns/148)	MSP	[45]
*hMLH1*	Tissue	21 (ns/243)	not tested	97 (ns/148)	MSP	[45]
*MGMT*	Tissue	39 (ns/243)	14 (ns/64)	96 (ns/148)	MSP	[45]
*RARbeta2*	Tissue	24 (ns/243)	not tested	100 (148/148)	MSP	[45]
*RASSF2A*	Tissue	58 (ns/243)	37 (ns/64)	100 (148/148)	MSP	[45]
*Wif-1*	Tissue	74 (ns/243)	32 (ns/64)	98 (ns/148)	MSP	[45]
*APC*, *MGMT*, *RASSF2A*, *Wif-1*	Plasma	87 (ns/243)	75 (ns/64)	92 (ns/148)	MSP	[45]
*RARB2*, *p16*^*INK4a*^, *MGMT*, *APC*	Tissue	ns (ns/12)	ns (ns/20)	no controls	MSP	[49]
*RARB2*, *p16*^*INK4a*^, *MGMT*, *APC*	Stool	75 (9/12)	60 (12/20)	no controls	MSP	[49]
*RARB2*, *p16*^*INK4a*^, *MGMT*, *APC*	Tissue	77 (20/26)	75 (18/20)	100 (20/20)	MS-MCA	[49]
*RARB2*, *p16*^*INK4a*^, *MGMT*, *APC*	Stool	62 (16/26)	40 (8/20)	100 (20/20)	MS-MCA	[49]

*ns*, not specified; *QuARTS*, quantitative allele-specific real-time target and signal amplification; *MSP*, methylation-specific PCR; *RRBS*, reduced representation bisulfite sequencing; *qMSP*, quantitative methylation-specific PCR; *MS-MCA*, methylation-specific melting curve analysis

When investigating the case-by-case relationship for single methylation markers analyzed in more than one type of material from the same patient, we found a very good agreement between tissue and other materials for four markers (*CDH4* in the blood, *ERCC1* in the blood, *p16*^*INK4a*^ in the blood, and *SPG20* in stool) (Table 3). Most of the markers (12 of 21) showed marginal reproducibility (*k* < 0.4) between methylation status in tissue and other materials (Table 3).

**Table 3:** Case-by-case relationship for single methylation markers in > 1 type of material from the same individual (other= blood (bold) or stool (italics) or urine (bold-italics))

***Gene***	**tissue+** **other+**	**tissue+** **other-**	**tissue-other+**	**tissue-other-**	**McNemar**	**k**	**SE**	**p**	**Reference**
*BCAT1*	**42**	**46**	**0**	**2**	< 0.001	0.039	0.027	0.181	[22]
*CDH4*	**10**	**2**	**0**	**9**	0.500	0.811	0.125	**< 0.001**	[50]
*CDH4*	*19*	*19*	*0*	*16*	<0.001	0.372	0.090	< 0.001	[51]
*DAPK*	**3**	**11**	**0**	**4**	0.001	0.108	0.076	0.310	[52]
*EFHD1*	**15**	**4**	**4**	**1**	1.000	-0.011	0.202	0.959	[32]
*ERCC1*	**34**	**0**	**3**	**13**	0.250	0.855	0.080	**< 0.001**	[30]
*GATA5*	*13*	*19*	*0*	*22*	< 0.001	0.358	0.091	0.001	[51]
*HLTF*	**10**	**14**	**1**	**29**	0.001	0.405	0.111	0.001	[53]
*HPP1*	**28**	**22**	**0**	**4**	< 0.001	0.159	0.075	0.031	[53]
*IKZF1*	**43**	**36**	**0**	**12**	< 0.001	0.226	0.063	0.001	[22]
*MGMT*	**29**	**5**	**0**	**16**	0.063	0.788	0.088	**< 0.001**	[30]
*OSMR*	**11**	**11**	**2**	**1**	0.022	-0.073	0.132	0.588	[38]
*OSMR*	**30**	**8**	**0**	**2**	0.008	0.273	0.158	0.012	[35]
*p16*^*INK4a*^	**11**	**0**	**0**	**7**	1.000	1.000	0	**< 0.001**	[54]
*p16*^*INK4a*^	**8**	**5**	**0**	**8**	0.063	0.549	0.157	**0.005**	[55]
*p16*	**7**	**1**	**0**	**3**	1.000	0.792	0.194	**0.007**	[56]
*p16*	**13**	**31**	**0**	**50**	< 0.001	0.308	0.073	< 0.001	[57]
*PCDH10*	**42**	**21**	**0**	**4**	< 0.001	0.193	0.086	0.008	[58]
*PPP1R3C*	**17**	**5**	**2**	**0**	0.453	-0.135	0.074	0.449	[32]
*RASSF1A*	**2**	**1**	**0**	**6**	1.000	0.727	0.247	**0.023**	[59]
*SEPT9*	**58**	**12**	**0**	**15**	< 0.001	0.630	0.092	<0.001	[58]
*SEPT9*	**30**	**3**	**1**	**0**	0.250	0.370	0.270	**0.005**	[37]
*SEPT9*	**28**	**7**	**0**	**5**	0.016	0.500	0.149	< 0.001	[35]
*SFRP1*	**19**	**4**	**1**	**1**	0.375	0.194	0.229	0.269	[38]
*SFRP2*	*4*	*3*	*1*	*0*	0.625	-0.231	0.192	0.408	[40]
*SPG20*	*77*	*5*	*0*	*14*	0.063	0.818	0.078	**< 0.001**	[42]
*VIM*	***12***	***5***	***3***	***0***	0.727	-0.231	0.093	0.278	[44]

*k*, Cohen’s kappa; *SE*, standard error of *k*

## Discussion

The present systematic review identified 51 publications analyzing cell-free DNA methylation markers in more than one type of material from the same individual. The markers analyzed in these studies are well known from previous studies in tissue, blood, and stool [11–16]. We will here discuss the potential role of each of the identified markers, as contributors to a panel for screening and follow-up of CRC (i.e., sensitivity and specificity for early detection, prognostic value, detection of residual disease and recurrence, ability to reflect ongoing oncologic treatment, and detectability in more than one type of material, i.e., blood (b), stool (s), urine (u), and tumor tissue (t)).

Bone morphogenetic protein 3 (BMP3) is a member of the transforming growth factor-beta (TGFβ) superfamily of cytokines, binding to cell-surface receptors, activating a cascade of cell signaling, ultimately regulating the transcription of SMAD4 target genes to achieve growth suppression [60]. Downregulation of the *BMP3* tumor suppressor gene is an early and frequent event in colorectal cancer [60]. In the present systematic review, methylation of *BMP3* was identified in 75% of plasma samples and 82% of tissue samples from patients with colorectal cancer [31]. However, methylation of *BMP3* was also detected in 11 of the 37 plasma samples from healthy controls referred to colonoscopy with no evidence of CRC or other cancer [31]. Tissue from healthy controls was not analyzed. *BMP3* has also been analyzed in other studies where the sensitivity and specificity for CRC in tissue were 57% and 93%, respectively [61], where the sensitivity and specificity for CRC in plasma varied from 29 to 40% and 89 to 94%, respectively [62, 63], and where methylation in stool DNA varied from 40 to 100% in CRC and 33 to 70% in advanced adenoma samples [64, 65]. *BMP3* is one of two methylation markers (the other is *NDRG4*) included in the commercially available fecal Cologuard® test, approved by the FDA in 2014 as a colon cancer screening test [46, 66].

*EFHD1* (EF-hand domain family member D1) encodes a calcium-binding protein involved in mitosis, synaptic transmission, and cytoskeletal rearrangement [67]. In the present systematic review, methylation of *EFHD1* had 79% sensitivity and 22% specificity for CRC in plasma [32]. When combined with the analysis of *PPP1R3C* methylation, sensitivity was 53% and specificity reached 96% [32]. No patients with adenoma were included in this study. We have not found other studies on *EFHD1* and its value as a potential biomarker for CRC is still unclear.

The *ITGA4* gene encodes a membrane protein (integrin alpha 4) and is considered a risk marker for inflammation-associated colon cancer [68]. One study identified in the present systematic review found this marker to have 80% sensitivity and 100% specificity for CRC in stool, and methylated *ITGA4* was found in 89% of CRC tissue samples and 88% of adenoma tissue samples [33]. In a stool only study, the sensitivity for adenoma was 29% and the specificity was 69% [69]. Another stool only study found 70% sensitivity and 97% specificity for CRC when investigating a panel combining *ITGA4*, *SFRP2*, and *p16* [70].

The N-Myc downstream-regulated gene 4 (*NDRG4*) plays a role in cell growth and differentiation and is a putative tumor suppressor shown to be downregulated in colorectal cancer [71]. In the present systematic review, methylation of *NDRG4* had 76% sensitivity for CRC in stool and 73% in urine [34, 72]. No patients with adenomas and no healthy controls were included; hence, no sensitivity for adenoma and no specificity were reported [34, 72]. A study on cell-free DNA in plasma found methylation of *NDRG4* in only 18 of 193 patients with CRC (sensitivity 9%) but in none of the healthy controls (specificity 100%) [62]. In two sets of samples from different individuals, *NDRG4* methylation analysis in stool found the sensitivity for CRC to be 53 to 61% and the specificity to be 93 to 100% [71]. Methylation was detected in 70 to 86% of CRC tissues as compared with 4% in noncancerous colon mucosa [71]. Despite *NDRG4* being included in the Cologuard® test, there are only few studies addressing its role as a potential biomarker.

The oncostatin M receptor gene (*OSMR*) encodes a subunit of both the oncostatin M (OSM) receptor type II and the interleukin-31 receptor and transduce signals with pro- or anti-proliferative functions. *OSMR* promoter methylation has been identified in tissue and stool samples from patients with CRC. Two of the included studies confirmed that *OSMR* methylation is a common epigenetic event in colorectal cancer [35, 38], but one study also revealed low concordance (48%) and specificity (33%) when comparing matched plasma and tumor tissue samples [38].

The protein phosphatase 1 regulatory subunit 3C (PPP1R3C) modulates glycogen metabolism, and methylation of the *PPP1R3C* gene has been proposed to play a critical role in colorectal cancer. One of the studies included in this review found that methylated *PPP1R3C* had 92% sensitivity for the detection of stage I CRC [32]. However, the sample size of this study was small and its value as a potential biomarker, especially for early-stage CRC cancer, needs to be further investigated.

*SEPT9* encodes a GTP-binding protein involved in cell proliferation and migration, cytokinesis, and angiogenesis [1]. Methylation of *SEPT9* is the most extensively studied single methylated marker for colorectal cancer. There are two commercialized assays already in clinical use as blood tests. These are ColoVantage® (sensitivity 90%) [73] and Epi proColon 2.0 (sensitivity 66–81% and specificity 96–99%) [74, 75]. However, the sensitivity in the blood for advanced adenomas has been < 10% in several studies [76–78]. In the present systematic review, the most well-designed studies generally found a good correlation between the methylation status of *SEPT9* in plasma and tissue for CRC patients and also for healthy controls [35, 36, 79, 80]. In patients with adenomas the impression is that even though patients have methylated *SEPT9* in tissue, only one-third of them have detectable methylated *SEPT9* in plasma [37]. The reason for this discrepancy remains unknown.

The secreted frizzled-related protein 1 and 2 genes (*SFRP1* and *SFRP2*) encode antagonists of the Wnt signaling pathway, acting as tumor suppressors. Altered methylation of these genes has been reported in colorectal cancer tissue [81]. Aberrant promoter methylation of *SFRP2* is associated with poor survival of colorectal cancer [39, 82]. Two well-designed studies found the sensitivity of *SFRP2* for CRC to be above 85% in stool and ranging from 46 to 62% for adenoma, the specificity was above 90%, and the findings corresponded well with the findings in tissue for the same patients [39, 40]. In studies of only one type of material, Rasmussen et al. found that the sensitivity of *SFRP2* for CRC to be 21% for CRC and the specificity was 82% in plasma [62]. In stool only studies, sensitivity for CRC was 63–94%, sensitivity for adenomas was 46–92%, and specificity was 32–100% [83–87]. As mentioned previously, the combination of *SFRP2* with other markers, such as *VIM*, *RASSF1A*, *TMEFF2*, *MGMT*, *ITGA4*, and *p16*, has provided promising results [70, 72, 85, 87, 88].

The spastic paraplegia 20 gene (*SPG20*) encodes Spartin, a multifunctional protein found to be involved in intracellular epidermal growth factor receptor trafficking [89]. In the present systematic review, we identified two studies investigating this biomarker in more than one type of material, although none of these studies included adenomas. Rezvani et al. found methylated *SPG20* in 94% of CRC tissues and correspondingly in 81% of the plasma samples from these patients [41]. The specificity in tissue samples was 99% and in plasma samples 97% [41]. Zhang et al. found methylated *SPG20* in 85% of CRC tissues and correspondingly in 80% of stool samples from these patients [42]. No tissue samples from healthy controls were investigated, but the specificity in the stool was 100% [42]. In a study carried out in plasma only, the sensitivity of *SPG20* was 16% for CRC and the specificity was 88%, whereas the sensitivity for adenomas was not investigated [62].

The tissue factor pathway inhibitor 2 gene (*TFPI2*) encodes a serine protease inhibitor that decreases the activities of several enzymes, thereby protecting the extracellular matrix from degradation and inhibiting in vitro colony formation and proliferation [68]. Loss of *TFPI2* function has been associated with pro-invasiveness and methylation of *TFPI2* is considered an independent prognostic factor for CRC as it has been associated with later stages of carcinogenesis and advanced colorectal cancer [68, 90]. *TFPI2* has been demonstrated to be completely unmethylated in tissue from healthy controls, whereas methylation frequency increases with progression into inflamed colon tissue or CRC [68]. In the present review, two studies investigating *TFPI2* methylation in stool and corresponding tissues found methylation of *TFPI2* in CRC tissues in 89% and 99% of the samples, respectively, and the sensitivity in stool was 80% and 76%, respectively [33, 43]. Specificity for CRC in stool was 100% and 93%, respectively [33, 43]. Glöckner et al. also found methylated *TFPI2* in three of the tissue samples from healthy controls (6%) and in 55 of 56 adenoma samples (98%) [43]. However, the sensitivity of *TFPI2* methylation in adenoma stool samples was only 21% [43]. In a study of plasma, the CRC sensitivity of *TFPI2* methylation was only 7%, no adenomas were included, and the specificity was 98% [62]. In a study of stool, the sensitivity for CRC was 68%, the specificity was 100%, whereas the sensitivity for adenoma was 35% [91, 92].

*VIM* encodes a protein of the cytoskeleton. *VIM* is considered a biomarker of mesenchymal-derived cells and cancer cells undergoing epithelial-mesenchymal transition (EMT) during invasion and metastasis, and promoter methylation of *VIM* has been documented in CRC [1]. ColoSure® is a fecal single-marker test used in combination with colonoscopy. The test detects methylated *VIM* as a marker for CRC (sensitivity38–88%) [1, 93]. In paired samples from patients with CRC and/or adenomas, the share with detectable methylated *VIM* was 33% in stool, 4% in serum, and 8% in urine and the specificity was 100% in all materials [94]. In one study, where 83% of CRC cases had methylated *VIM* in tissue samples, 55% also had methylated *VIM* in stool [95]. In another study, where 44% of CRC cases had methylated *VIM* in tissue, 40% also had methylated *VIM* in stool [33]. Gerecke et al. also found that 72% of patients with adenomas had methylated *VIM* in adenoma tissue [33]. In Song et al.’s study, 85% of CRC cases had methylated *VIM* in tissue samples and 75% also had methylated *VIM* in urine [44]. When combining analysis of *SFRP2* and *VIM*, methylation in at least one of these was found in 92% of CRC and 94% of adenoma tissues and correspondingly in 89% and 85% of stool samples [72]. *VIM* methylation is extensively studied in unpaired samples. The sensitivity for CRC detection by *VIM* methylation in the blood is 18–59% and the specificity is 63–93% [62, 96, 97]. The sensitivity for CRC in stool is 38–85%, and the sensitivity and specificity for adenoma are 75–90% and 82–100 % respectively [98–103].

One plasma panel consisting of the methylation markers *APC*, *MGMT*, *RASSF2A*, and *WIF1* was identified by the present systematic review as promising, by reporting 86% sensitivity and 92% specificity for CRC [45]. Methylation in corresponding fresh-frozen tissue samples was found to be 27% for *APC* (specificity 97%), 39% for *MGMT* (specificity 96%), 58% for *RASSF2A* (specificity 100%), and 74% for *WIF1* (specificity 98%) [45]. Methylation of *APC* leads to activation of growth-promoting genes, methylation of *MGMT* impairs the elimination of DNA alkylation adducts which may lead to mutation, methylation of the tumor suppressor *RASSF2* alters the regulation of the cytoskeleton and apoptosis, and methylation of *WIF1* results in reduced blocking of the Wnt signaling pathway [45].

We also identified two stool panels as the most promising panels tested in more than one type of material from the same individual. One was the panel consisting of methylated *BMP3/NDRG4/VIM/TFPI2/*mutant *KRAS*, β-actin, and Hb-level (and later refined and commercialized as Cologuard®), which in stool detected CRC with 87% sensitivity, adenoma with 82% sensitivity, and demonstrated a specificity of 93% [46, 48, 66]. Corresponding plasma samples were tested for methylated *SEPT9* and the reported sensitivity for CRC was 60%, adenoma 14%, and the specificity was 73% [48]. The second stool panel identified was one consisting of *RARB2*, *p16*, *MGMT*, and *APC.* This panel detected CRC with 75% sensitivity, adenomas with 60% sensitivity, and the specificity was 100% [49]. To our knowledge, no other research groups have reproduced these findings and the findings have not led to a commercialized test.

Our overall impression is that the quality of the included studies was variable. With reference to QUADAS; many studies do not clearly classify the patients with colorectal cancer (some also include patients with adenomas and polyps), many have a sub-optimal or mixed “control group” (self-declared healthy controls or the control group including inflammatory bowel disease, colitis, polyps), many do not report blinding (i.e., it is not known whether the researcher who performed the methylation analysis knew whether the patient was a healthy control or a cancer patient), and many studies fail to report important findings (“not specified” in Supplementary Tables 1 and 2). To determine the strength of each methylated marker, it would be useful to aggregate the findings from single studies into a meta-analysis. Currently, this is not possible due to inconsistency in the reporting. To ensure comparability and future aggregation of findings, we suggest that future studies report their findings according to Table 4.

**Table 4:** Standardized reporting from studies of methylated markers in cell-free DNA

Gene marker	Methylation in material*				Sensitivity CRC			Sensitivity adenoma			Specificity**			Method***
	Tissue+ other+	Tissue+ other−	Tissue− other+	Tissue– other−	%	*n*/*N*	CI	%	*n*/*N*	CI	%	*n*/*N*	CI	
Single marker X	*n*	*n*	*n*	*n*										
Single marker Y	*n*	*n*	*n*	*n*										
Panel of markers XY	*n*	*n*	*n*	*n*										

*N and n*, absolute numbers; *CI*, 95% confidence interval with upper and lower limits

*Specification of the type of other material analyzed (in addition to tissue). Examples: stool, plasma, serum, urine

**Specification of the type of “control group.” Examples: colonoscopy-verified healthy controls, self-declared healthy controls, age-matched colonoscopy-verified healthy controls, etc.

***Specification of the method used. Examples: *MSP*, methylation-specific PCR; *qMSP*, quantitative methylation-specific PCR; *MA*, microarray analysis; *MS-MCA*, methylation-specific melting curve analysis; *MSRE-PCR*, methylation-sensitive restriction enzyme and multiplex PCR; *SEQ*, sequencing; *RRBS*, reduced representation bisulfite sequencing

During the present review, we found that many of the studies claiming to report methylation in so-called paired samples do not analyze corresponding materials from the same individual. In addition, few of the studies reported the absolute numbers needed to calculate sensitivity and sensitivity. For the studies reporting the absolute numbers of methylation status in tissue and corresponding other materials from single individuals, we performed a case-by-case analysis (Table 3). The correspondence of methylation status in different materials from the same individual was promising for *CDH4*, *ERCC1*, *p16*^*INK4a*^, and *SPG20*. However, for most of the methylation markers and in most of the corresponding materials investigated (16 of 27), only a marginal reproducibility of methylation status was found. This inconsistency between “liquid biopsies” and tissue biopsies is confusing and needs to be addressed by analyzing methylation markers in several different materials from the same individual. And, this needs to be done both for patients with CRC, patients with adenoma, and healthy controls.

In the publications reviewed here, we rediscover some of the known technical challenges to the practical and clinical application of methylated cell-free DNA markers [58]. To date, there are still no standards agreed upon for analysis of methylated cell-free DNA and among the included publications, there is considerable variation regarding the methods used to extract and quantify cell-free DNA. The methods for sample processing and analysis also vary a lot, and many of the studies do not address or report upon pre-analytical and analytical issues. A study on optimization of the yield and utility of circulating cell-free DNA showed that time from blood sampling to plasma separation was extremely significant, whereas the temperature of the blood sample before plasma separation (kept on ice or at room temperature) did not affect the amount of cell-free DNA significantly [104]. One report has stated that DNA levels in plasma stored at − 80 °C declines by 30% per year [105]. The cell-free DNA methylation pattern may be affected by demographic and lifestyle characteristics (i.e., age, race, gender, smoking, alcohol consumption), diet (i.e., folate, green tea, phytoestrogen), environmental exposures (i.e., arsenic, cadmium), and disease status [106]. Only a few of the studies take such factors into account when analyzing and reporting their results. The small sample sizes, selective and incomplete reporting, and lack of independent validation of promising biomarkers are factors hampering the implementation of potential biomarkers into clinical use [107]. The use of sensitive technologies, unbiased optimization, and standardization of commonly used methods is utterly important to enable validation of the findings from promising biomarker discovery studies [108].

## Conclusion

The identified studies analyzing methylated DNA markers in paired samples from the same individuals generally included few patients, reported only some of the variables of interest and investigated only some of the markers of interest. Hence, a meta-analysis was not possible at this point. Larger, prospective studies need to be designed to overcome the challenge of discordant detection of markers in tissue and liquid biopsies. To improve comparability and to facilitate aggregation of the evidence base regarding possible DNA methylation markers of colorectal cancer, we suggest that this type of study use a standardized format when reporting their findings.

## Electronic supplementary materials

ESM 1 (DOCX 63 kb)

ESM 2 (DOCX 21 kb)

## Data Availability

Not applicable.
