# Supplementary material for: DNA methylation markers detected in blood, stool, urine, and tissue in colorectal cancer: a systematic review of paired samples
Source: Int J Colorectal Dis. 2020 Oct 6;36(2):239–51. doi: 10.1007/s00384-020-03757-x (PMC7801356; doi:10.1007/s00384-020-03757-x)
Supplement: Supplementary file 1 — (DOCX 63 kb) [file 384_2020_3757_MOESM1_ESM.docx]

| **Supplementary Table 1. Single markers in >1 material** | | | | | | |  |
| --- | --- | --- | --- | --- | --- | --- | --- |
| ***Gene*** | **Specimen** | **Sensitivity CRC % (*n*)** | **Sensitivity adenoma % (*n*)** | **Specificity**  **% (*n*)** | **Method** | **Author** | **Year** |
| *ALX4* | stool | 11 (9/90) | | 99 (155/157) | qMSP | Amiot | 2014 |
| *ALX4* | serum | 23 (11/48) | | 100 (14/14) | qMSP | Amiot | 2014 |
| *ALX4* | urine | 15 (7/48) | | 100 (14/14) | qMSP | Amiot | 2014 |
| *ALX4* | plasma | 48 (87/182) | no adenomas | 94 (159/170) | qMSP | He | 2010 |
| *ALX4* | tissue | 56 (71/127) | no adenomas | 90 (108/120) | qMSP | He | 2010 |
| *BCAT1* | tissue | 98 (89/91) | no adenomas | no controls | MSP | Symonds | 2018 |
| *BCAT1* | plasma | 47 (43/91) | no adenomas | no controls | MSP | Symonds | 2018 |
| *BMP3* | plasma | 75 (44/59) | no adenomas | 70 (26/37) | MSP | Ashoori | 2018 |
| *BMP3* | tissue | 81 (24 /30) | no adenomas | ns (ns/37) | MSP | Ashoori | 2018 |
| *CDH4* | blood | 70 (32/46) | ns | 100 (17/17) | MSP | Miotto | 2004 |
| *CDH4* | tissue | 57 (12/21) | 100 (10/10) | 100 (10/10) | MSP | Miotto | 2004 |
| *CDH4* | tissue | 70 (38/54) | no adenomas | no controls | MSP | Nishioka | 2015 |
| *CDH4* | stool | 35 (19/54) | no adenomas | no controls | MSP | Nishioka | 2015 |
| *DAPK* | tissue | 55 (67/122) | no adenomas | no controls | MSP | Yamaguchi | 2003 |
| *DAPK* | serum | 21 (3/14) | no adenomas | no controls | MSP | Yamaguchi | 2003 |
| *EFHD1* | tissue | 79 (19/24) | no adenomas | ns (ns/ns) | MSP | Takane | 2014 |
| *EFHD1* | plasma | 79 (19/24) | no adenomas | 78 (75/96) | MSP | Takane | 2014 |
| *ER alpha* | blood | ns (ns/27) | ns (ns/30) | ns (ns/57) | qMSP | Ally | 2009 |
| *ER alpha* | tissue | ns (ns/28) | ns (ns/35) | ns (ns/76) | qMSP | Ally | 2009 |
| *ERCC1* | tissue | 74 (37/50) | 19 (8/43) | no controls | MSP | Shalaby | 2018 |
| *ERCC1* | blood | 60 (30/50) | 7 (3/43) | no controls | MSP | Shalaby | 2018 |
| *GATA5* | tissue | 59 (32/54) | no adenomas | no controls | MSP | Nishioka | 2015 |
| *GATA5* | stool | 24 (13/54) | no adenomas | no controls | MSP | Nishioka | 2015 |
| *HLTF* | tissue | 44 (24/54) | no adenomas | no controls | qMSP | Philipp | 2012 |
| *HLTF* | serum | 46 (11/24) | no adenomas | no controls | qMSP | Philipp | 2012 |
| *HPP1* | tissue | 93 (50/54) | no adenomas | no controls | qMSP | Philipp | 2012 |
| *HPP1* | serum | 56 (28/50) | no adenomas | no controls | qMSP | Philipp | 2012 |
| *IKZF1* | tissue | 87 (79/91) | no adenomas | no controls | MSP | Symonds | 2018 |
| *IKZF1* | plasma | 47 (43/91) | no adenomas | no controls | MSP | Symonds | 2018 |
| *ITGA4* | tissue | 89 (8/9) | 88 (44/50) | no controls | nMSP | Gerecke | 2012 |
| *ITGA4* | stool | 80 (4/5) | no adenomas | 100 (5/5) | nMSP | Gerecke | 2012 |
| *MGMT* | blood | 58 (29/50) | 5 (2/43) | no controls | MSP | Shalaby | 2018 |
| *MGMT* | tissue | 80 (40/50) | 14 (6/43) | no controls | MSP | Shalaby | 2018 |
| *NDRG4* | tissue | 81 (68/84) | no adenomas | 92 (77/84)d | nMSP | Xiao | 2015 |
| *NDRG4* | stool | 76 (64/84) | no adenomas | 89 (ns/ns) | nMSP | Xiao | 2015 |
| *NDRG4* | blood | 55 (46/84) | no adenomas | 78 (ns/ns) | nMSP | Xiao | 2015 |
| *NDRG4* | urine | 73 (61/84) | no adenomas | 85 (ns/ns) | nMSP | Xiao | 2015 |
| *OSMR* | tissue | 88 (22/25) | ns | ns (ns/56) | MSP | Bedin | 2017 |
| *OSMR* | plasma | 52 (13/25) | 17 (3 /18) | 86 (31/36) | MSP | Bedin | 2017 |
| *OSMR* | plasma | 75 (30/40) | no adenomas | no controls | MSP | Yuan | 2016 |
| *OSMR* | tissue | 95 (38/40) | no adenomas | no controls | MSP | Yuan | 2016 |
| *p16 ink4A* | tissue | 21 (14/66) | no adenomas | no controls | MSP | Bazan | 2006 |
| *p16 ink4A* | plasma | 17 (3/18) | no adenomas | no controls | MSP | Bazan | 2006 |
| *p16 ink4A* | plasma | 61 (11/18) | no adenomas | ns (ns/20) | MSP | Frattini | 2008 |
| *p16 ink4A* | tissue | 100 (11/11) | no adenomas | ns (ns/20) | MSP | Frattini | 2008 |
| *p16* | tissue | 53 (31/58) | no adenomas | no controls | MSP | Lecomte | 2002 |
| *p16* | plasma | 68 (21/31) | no adenomas | no controls | MSP | Lecomte | 2002 |
| *p16* | tissue | 59 (99/168) | no adenomas | 100 (168/168)d | qMSP | Nakayama | 2007 |
| *p16* | serum | ns (ns/99) | no adenomas | 100 (30/30) | qMSP | Nakayama | 2007 |
| *p16 ink4A* | tissue | 62 (13/21) | no adenomas | 100 (21/21)d | qMSP | Nakayama | 2011 |
| *p16 ink4A* | blood | 62 (8/13) | no adenomas | 100 (20/20) | qMSP | Nakayama | 2011 |
| *p16* | tissue | 73 (8/11) | no adenomas | no controls | MSP | Nakayama | 2003 |
| *p16* | serum | 88 (7/8) | no adenomas | no controls | MSP | Nakayama | 2003 |
| *p16* | tissue | 47 (44/94) | no adenomas | no controls | MSP | Nakayama | 2002 |
| *p16* | serum | 30 (13/44) | no adenomas | no controls | MSP | Nakayama | 2002 |
| *PCDH10* | tissue | 94 (63/67) | no adenomas | 90 (60/67)d | qMSP | Danese | 2013 |
| *PCDH10* | plasma | 63 (42/67) | no adenomas | no controls | qMSP | Danese | 2013 |
| *PPP1R3C* | tissue | 92 (22/24) | no adenomas | ns (ns/ns) | MSP | Takane | 2014 |
| *PPP1R3C* | plasma | 79 (19/24) | no adenomas | 81 (78/96) | MSP | Takane | 2014 |
| *PRIMA1* | plasma | 81 (38/47) | 70 (26/37) | ns (ns/37) | MSP | Bartak | 2017 |
| *PRIMA1* | tissue | ns (ns/10) | ns (ns/11) | ns (ns/11) | MSP | Bartak | 2017 |
| *RASSF1A* | tissue | 33 (3/9) | no adenomas | 89 (8/9)d | MSP | Wang_Detect | 2008 |
| *RASSF1A* | serum | 22 (2/9) | 7 (2/30) | 100 (30/30) | MSP | Wang_Detect | 2008 |
| *SDC2* | plasma | 89 (42/47) | 81 (30/37) | ns (ns/37) | MSP | Bartak | 2017 |
| *SDC2* | tissue | ns (ns/10) | ns (ns/11) | ns (ns/11) | MSP | Bartak | 2017 |
| *SEPT9* | stool | 100 (11/11) | no adenomas | 100 (3/3) | MSP | Aleksandra | 2015 |
| *SEPT9* | plasma | 55 (6/11) | no adenomas | 100 (3/3) | MSP | Aleksandra | 2015 |
| *SEPT9* | tissue | 93 (13/14) | no adenomas | 100 (26/26)a | pSEQ | Carmona | 2013 |
| *SEPT9* | stool | 20 (7/35) | no adenomas | ns | pSEQ | Carmona | 2013 |
| *SEPT9* | tissue | 82 (70/85) | no adenomas | no controls | qMSP | Danese | 2015 |
| *SEPT9* | plasma | 68 (58/85) | no adenomas | no controls | qMSP | Danese | 2015 |
| *SEPT9* | plasma | 75 (136/182) | no adenomas | 97 (164/170) | qMSP | He | 2010 |
| *SEPT9* | tissue | 78 (99/127) | no adenomas | 97 (116/120) | qMSP | He | 2010 |
| *SEPT9* | tissue | 97 (33/34) | 100 (26/26) | 96 (23/24) | MSP | Toth_Detection | 2014 |
| *SEPT9* | plasma | 88 (30/34) | 31 (8/26) | 92 (22/24) | MSP | Toth_Detection | 2014 |
| *SEPT9* | plasma | 70 (28/40) | no adenomas | no controls | MSP | Yuan | 2016 |
| *SEPT9* | tissue | 88 (35/40) | no adenomas | no controls | MSP | Yuan | 2016 |
| *SFRP1* | plasma | 85 (40/47) | 89 (33/37) | ns (ns/37) | MSP | Bartak | 2017 |
| *SFRP1* | tissue | ns (ns/10) | ns (ns/11) | ns (ns/11) | MSP | Bartak | 2017 |
| *SFRP1* | tissue | 92 (23/25) | ns (ns/22) | ns (ns/56) | MSP | Bedin | 2017 |
| *SFRP1* | plasma | 80 (20/25) | 17 (3/18) | 92 (33/36) | MSP | Bedin | 2017 |
| *SFRP2* | plasma | 72 (34/47) | 84 (31/37) | ns (ns/37) | MSP | Bartak | 2017 |
| *SFRP2* | tissue | ns (ns/10) | ns (ns/11) | ns (ns/11) | MSP | Bartak | 2017 |
| *SFRP2* | tissue | 88 (149/169) | 65 (41/63) | 100 (30/30) | MSP | Tang | 2011 |
| *SFRP2* | stool | 84 (142/169) | 46 (29/63) | 93 (28/30) | MSP | Tang | 2011 |
| *SFRP2* | serum | 67 (113/169) | 6 (4 /63) | 100 (30/30) | MSP | Tang | 2011 |
| *SFRP2* | tissue | 91 (63/69) | 79 (27/34) | 100 (30/30)b | MSP | Wang_Hyperm | 2008 |
| *SFRP2* | stool | 87 (60/69) | 62 (21/34) | 93 (28/30) | MSP | Wang_Hyperm | 2008 |
| *SPG20* | tissue | 94 (30/32) | no adenomas | 99 (ns/32)d | qMSP | Rezvani | 2017 |
| *SPG20* | plasma | 81 (30/37) | no adenomas | 97 (ns/37) | qMSP | Rezvani | 2017 |
| *SPG20* | tissue | 85 (82/96) | no adenomas | no controls | MSP | Zhang | 2013 |
| *SPG20* | stool | 80 (77/96) | no adenomas | 100 (30/30) | MSP | Zhang | 2013 |
| *TFPI2* | tissue | 89 (8/9) | 64 (32/50) | no controls | nMSP | Gerecke | 2012 |
| *TFPI2* | stool | 80 (4/5) | no adenomas | 100 (5/5) | nMSP | Gerecke | 2012 |
| *TFPI2* | tissue | 99 (114/115) | 98 (55/56) | 94 (45/48) | MSP | Glökner | 2009 |
| *TFPI2* | stool | 76 (50/66) | 21 (4/19) | 93 (28/30)b | qMSP | Glökner | 2009 |
| *TMEFF2* | plasma | 71 (129/182) | no adenomas | 95 (162/170) | qMSP | He | 2010 |
| *TMEFF2* | tissue | 75 (95/127) | no adenomas | 94 (113/120) | qMSP | He | 2010 |
| *VIM* | stool | 33 (29/90) | | 100 (0/157) | qMSP | Amiot | 2014 |
| *VIM* | serum | 4 (2/48) | | 100 (14/14) | qMSP | Amiot | 2014 |
| *VIM* | urine | 8 (4/48) | | 100 (14/14) | qMSP | Amiot | 2014 |
| *VIM* | tissue | 83 (10/12) | no adenomas | 86 (19/22)a | pSEQ | Carmona | 2013 |
| *VIM* | stool | 55 (18/33) | no adenomas | ns | pSEQ | Carmona | 2013 |
| *VIM* | tissue | 44 (4/9) | 72 (36/50) | no controls | nMSP | Gerecke | 2012 |
| *VIM* | stool | 40 (2/5) | no adenomas | 100 (5/5) | nMSP | Gerecke | 2012 |
| *VIM* | tissue | 85 (17/20) | no adenomas | ns (ns/20) | qMSP | Song | 2012 |
| *VIM* | urine | 75 (15/20) | no adenomas | 90 (18/20) | qMSP | Song | 2012 |
| *WIF1* | stool | 19 (17/90) | | 99 (156/157) | qMSP | Amiot | 2014 |
| *WIF1* | serum | 33 (29/90) | | 99 (155/157) | qMSP | Amiot | 2014 |
| *WIF1* | urine | 27 (24/90) | | 99 (155/157) | qMSP | Amiot | 2014 |
| ns, not specified. MSP, methylation‐specific PCR; qMSP, quantitative methylation‐specific PCR; nMSP, nested methylation-specific PCR; pSEQ, pyrosequencing | | | | | | | |
|  | | | | | | | |
|  | |  |  |  |  |  |  |
| a the control group included healthy subjects | | | |  |  |  |  |
| b the control group were colonoscopy-verified healthy controls and small adenoma | | | | | | |  |
| c self-declared healthy | | |  |  |  |  |  |
| d normal mucosa in CRC-patient as "control group" | | | |  |  |  |  |
