# Supplementary material for: DNA methylation markers detected in blood, stool, urine, and tissue in colorectal cancer: a systematic review of paired samples
Source: Int J Colorectal Dis. 2020 Oct 6;36(2):239–51. doi: 10.1007/s00384-020-03757-x (PMC7801356; doi:10.1007/s00384-020-03757-x)
Supplement: Supplementary file 2 — (DOCX 21 kb) [file 384_2020_3757_MOESM2_ESM.docx]

**Supplementary Table 2. Markers tested as panels in >1 material**

| ***Genes*** | **Specimen** | **Sensitivity CRC % (*n*)** | **Sensitivity adenoma**  **% (*n*)** | **Specificity**  **% (*n*)** | **Method** | **Author** | **Year** |
| --- | --- | --- | --- | --- | --- | --- | --- |
| *BMP3, NDRG4, VIM, TFPI2, mutant KRAS, B-actin, Hb* | stool | 87 (26/30) | 82 (18/22) | 93 (43/46) | QuARTS | Ahlquist | 2012 |
| *SEPT9* | plasma | 60 (18/30) | 14 (3/22) | 73 (36/49) | MSP | Ahlquist | 2012 |
| *GPR75* | tissue | 52(22/42) | no adenomas | ns (ns/ns) | RRBS | Ashktorab | 2014 |
| *EID3* | tissue | 81 (34/42) | no adenomas | ns (ns/ns) | RRBS | Ashktorab | 2014 |
| *BMP3* | tissue | 57 (24/42) | no adenomas | ns (ns/ns) | RRBS | Ashktorab | 2014 |
| *BMP3, EID3, GAS7, GPR76* | blood | ns (ns/42) | no adenomas | ns (ns/42) | RRBS | Ashktorab | 2014 |
| *CACNAG1, NEUROG1, RUNX3, SOCS1, MLH1* | tissue | ns (ns/25) | no adenomas | no controls | MSP | Gonzales-Pons | 2013 |
| *CACNAG1* | plasma | 90 (18/20) | no adenomas | no controls | MSP | Gonzales-Pons | 2013 |
| *CDKN2A, RASSF1, HIN1, CCDN2, SFRP1, p16* | plasma | ns (ns/20) | no adenomas | ns (ns/16) | qMSP | Kadam | 2012 |
| *CDKN2A, RASSF1, HIN1, CCDN2, SFRP1, p17* | tissue | ns (ns/20) | no adenomas | ns (ns/16) | qMSP | Kadam | 2012 |
| *ESR1, MGMT, HPP1, p16^INK4a^, APC, MLH1* | tissue | ns | ns | ns | MSP | Belshaw | 2004 |
| *ESR1, MGMT, HPP1, p16^INK4a^, APC, MLH1* | stool | ns (ns/2) | ns (ns/10) | ns (ns/18) | MSP | Belshaw | 2004 |
| *EYA4, GRIA4, ITGA4, MAP3K12-AS1, MSC* | tissue | ns (ns/39) | no adenomas | ns (ns/50) | MSP | Barault | 2018 |
| *EYA4, GRIA4, ITGA4, MAP3K12-AS1, MSC* | plasma | ns (ns/39) | no adenomas | ns (ns/50) | MSP | Barault | 2018 |
| *p14* | tissue | 18 (ns/243) | not tested | 98 (ns/148) | MSP | Lee | 2009 |
| *p16* | tissue | 34 (ns/243) | not tested | 97 (ns/148) | MSP | Lee | 2009 |
| *APC* | tissue | 27 (ns/243) | 18 (ns/64) | 97 (ns/148) | MSP | Lee | 2009 |
| *DAPK* | tissue | 34 (ns/243) | not tested | 100 (148/148) | MSP | Lee | 2009 |
| *HLTF* | tissue | 32 (ns/243) | not tested | 98 (ns/148) | MSP | Lee | 2009 |
| *hMLH1* | tissue | 21 (ns/243) | not tested | 97 (ns/148) | MSP | Lee | 2009 |
| *MGMT* | tissue | 39 (ns/243) | 14 (ns/64) | 96 (ns/148) | MSP | Lee | 2009 |
| *RARbeta2* | tissue | 24 (ns/243) | not tested | 100 (148/148) | MSP | Lee | 2009 |
| *RASSF2A* | tissue | 58 (ns/243) | 37 (ns/64) | 100 (148/148) | MSP | Lee | 2009 |
| *Wif-1* | tissue | 74 (ns/243) | 32 (ns/64) | 98 (ns/148) | MSP | Lee | 2009 |
| *APC, MGMT, RASSF2A, Wif-1* | plasma | 87 (ns/243) | 75 (ns/64) | 92 (ns/148) | MSP | Lee | 2009 |
| *MAL* | serum | ns (ns/12) | no adenomas | 50 (10/20) | qMSP | Liu_Serum | 2013 |
| *SEPT9* | serum | ns (ns/15) | no adenomas | 90 (18/20) | qMSP | Liu_Serum | 2013 |
| *TAC1* | serum | ns (ns/17) | no adenomas | 30 (6/20) | qMSP | Liu_Serum | 2013 |
| *TAC1, SEPT9, MAL* | tissue | ns (ns/26) | no adenomas | no controls | qMSP | Liu_Serum | 2013 |
| *RARB2, p16 ^INK4a^, MGMT, APC* | tissue | ns (ns/12) | ns (ns/20) | no controls | MSP | Azuara | 2010 |
| *RARB2, p16 ^INK4a^, MGMT, APC* | stool | 75 (9/12) | 60 (12/20) | no controls | MSP | Azuara | 2010 |
| *RARB2, p16 ^INK4a^, MGMT, APC* | tissue | 77 (20/26) | 75 (18/20) | 100 (20/20) | MS-MCA | Azuara | 2010 |
| *RARB2, p16 ^INK4a^, MGMT, APC* | stool | 62 (16/26) | 40 (8/20) | 100 (20/20) | MS-MCA | Azuara | 2010 |
| *SFRP2, VIM* | stool | 89 (33/37) | 85 (22/26) | ns (ns/57) | MSP | Xiao | 2014 |
| *SFRP2, VIM* | tissue | 92 (34/37) | 92 (24/26) | 97 (61/63) | MSP | Xiao | 2014 |
| *SST, MAL, TAC1, SEPT9, EYA4, CRABP1, NELl1* | serum | ns (ns/162) | no adenomas | no controls | MSP | Liu_methylation | 2016 |
| *SST, MAL, TAC1, SEPT9, EYA4, CRABP1, NELl2* | tissue | ns (ns/162) | no adenomas | no controls | MSP | Liu_methylation | 2016 |
| *WiF1, NPY, PENK* | tissue | ns (ns/41) | no adenomas | no controls | MSP | Garrigou | 2016 |
| *WiF1, NPY, PENK* | plasma | ns (ns/41) | no adenomas | no controls | MSP | Garrigou | 2016 |
| *12 genes hypermethylated CpG sites* | blood | ns (ns/ns) | ns (ns/ns) | ns (ns/ns) | ns | Speight | 2013 |
| *12 genes hypermethylated CpG sites* | stool | ns (ns/ns) | ns (ns/ns) | ns (ns/ns) | ns | Speight | 2013 |

ns,not specified. QuARTS, quantitative allele-specific real-time target and signal amplification. MSP, methylation‐specific PCR. RRBS, reduced representation bisulfite sequencing. qMSP, quantitative methylation‐specific PCR. MS‐MCA, methylation‐specific melting curve analysis
